# Fabrication and Characterization of the Newly Developed Superalloys Based on Inconel 740

**DOI:** 10.3390/ma13102362

**Published:** 2020-05-21

**Authors:** Małgorzata Grudzień-Rakoczy, Łukasz Rakoczy, Rafał Cygan, František Kromka, Zenon Pirowski, Ondrej Milkovič

**Affiliations:** 1 Lukasiewicz Research Network-Krakow Institute of Technology, Zakopianska 73, 30-418 Cracow, Poland; zenon.pirowski@kit.lukasiewicz.gov.pl; 2 Faculty of Metals Engineering and Industrial Computer Science, AGH University of Science and Technology, Mickiewicza 30, 30-059 Cracow, Poland; lrakoczy@agh.edu.pl; 3 Consolidated Precision Products, Investment Casting Division, Hetmańska 120, 35-078 Rzeszow, Poland; Rafal.Cygan@cppcorp.com; 4 Institute of Materials Research, Slovak Academy of Sciences, Watsonova 47, 040 01 Košice, Slovakia; fkromka@saske.sk (F.K.); omilkovic@saske.sk (O.M.); 5 Faculty of Materials, Metallurgy and Recycling Technical, University of Košice, Letná 9, 042 00 Košice, Slovakia; 6 Institute of Experimental Physics, Slovak Academy of Sciences, Watsonova 47, 040 01 Košice, Slovakia

**Keywords:** Inconel 740, A-USC, tantalum, superalloy, investment casting

## Abstract

The chemical composition of standard Inconel 740 superalloy was modified by changes in the Al/Ti ratio (0.7, 1.5, 3.4) and addition of Ta (2.0, 3.0, 4.0%). Remelted Inconel 740 (A0) and nine variants with various chemical compositions were fabricated by lost-wax casting. The microstructure, microsegregation, phase transformation temperatures, thermal expansion coefficients and hardness of the superalloys were investigated by scanning electron microscopy, energy dispersive X-ray spectroscopy, differential scanning calorimetry, dilatometry and Vickers measurements. Typical dendritic microstructure was revealed with microsegregation of the alloying elements. Segregation coefficient k^i^ for Ti, Nb and Ta did not exceed unity, and so precipitates enriched mainly in these elements were found in interdendritic spaces. The Nb-rich blocky precipitates, MC carbides, MN nitrides, oxides, and fine γ’ was in all modified castings. Presence of other microstructural features, such as Ti-rich needles, eutectic γ-γ’ islands, small Al-rich and Cr-rich precipitates depended on the casting composition. The lowest solidus and liquidus temperatures were observed in superalloys with a high Al/Ti ratio. Consequently, in A7–A9 variants, the solidification range did not exceed 100 °C. In the A0 variant the difference between liquidus and solidus temperature was 138 °C. Hardness of all modified superalloys was at least 50% higher than for the remelted Inconel 740 (209 HV10).

## 1. Introduction

Electricity is presently a key pillar of mankind’s living and economic development. Despite various methods of electricity production, a significant part of the energy sector is still based on coal. Forecasts predict that this state will continue for the next decades, despite the continuously repeated economic crises in the oil & gas branch. The factor that can successfully mitigate the effects of such crises is the continuous improvement of power unit efficiency. This has prompted the power sector to increase the pressure and temperature of their steam boilers to reduce CO_2_, NO_x_, and SO_x_ emissions [1,2,3,4]. Due to environmental restrictions having increased in recent years, new advanced ultra-supercritical (A-USC) plants ensure presently higher efficiencies compared to conventional fossil-burning power plants. Increasing the efficiency from 37% to 46%, by increasing the temperature to 760 °C and pressure to 35 MPa, lead to a reduction of CO_2_ emissions by 22% [5,6,7]. Coal processing, both simple processes and those complex multistage, require equipment that includes castings able to operate at high-homologous temperatures and aggressive corrosive environments. Austenitic stainless steels currently used in boiler applications do not have adequate oxidation and creep resistance and cannot ensure sufficient, safe exploitation through thousands of hours. It is expected that new generation materials will guarantee excellent oxidation-resistance, strength, and microstructural stability during the harsh service conditions [5,8,9,10,11]. Service requirements of the A-USC boiler superheater and reheater for exposure to 700 °C also exceed the possibilities of widely used alloys like HR6W, GH2984, IN617, Haynes 230 and Nimonic 263 [1,12,13,14]. Wrought Inconel 740 (Alloy 740), a precipitation strengthened Ni-based superalloy, fulfilled the operating steam service aims of the European Thermie AD700 program for boiler tubing [15,16]. Inconel 740 was designed to meet rigorous requirements, namely: rupture life for 100 MPa at 750 °C: 100,000 h and metal loss due to corrosion not to exceed 2 mm after 200,000 h [17]. To develop the appropriate microstructure and strength required for use in A-USC operating environments, the alloy is subjected to solution and ageing heat treatment. Thanks to the balance of Al, Ti and Nb concentrations, the matrix is strengthened by 15–20% of L1_2_-ordered γ’ phase, MC and complex M_23_C_6_ carbides. In comparison to Nimonic 263, is has increased Cr and decreased Mo contents, respectively. Low content of Mo (around 0.5%) and lack of W ensure excellent coal ash resistance, which is one of the critical criteria for 700 °C. They favor the breakdown of protecting chromium oxide layers in contact with sulfate and chloride-containing ash deposits, thereby making some common alloys defenseless to pitting attack. The microstructure of Inconel 740 can consist of undesirable phases like silicon-rich G-phase and the DO_24_-ordered hexagonal η-Ni_3_Ti phase [15,18,19,20,21]. The US A-USC Consortium selected more requiring service parameters of 760 °C and 35 MPa. It stated that to improve its phase stability and heavy-section weldability, the modification of Inconel 740 composition is necessary. One of the well-recognized chemical composition changes to standard Inconel 740 was the 740H, in which Al/Ti ratio was increased with decreasing the Si, Nb and B contents. Thanks to this, it fulfills the design requirements for both the US and European A-USC projects [22,23,24,25]. 

The microstructure stability and strength of superalloys during long-term service depend strongly on the alloying elements and their concentrations. Alloying has always been the main design strategy for stabilizing phase compositions, microstructural constituents morphology, and mechanical properties like creep and fatigue resistance. Some investigations aimed at modifying the chemical composition of Inconel 740 to improve properties, including increasing γ’ phase stability and mechanical properties, counteraction of brittle phases’ formation and improvement of corrosion resistance, have been reported [5,26,27,28,29]. One alloying element, tantalum, present in all monocrystalline Ni-based superalloys, performs strengthening functions in both γ and the precipitating γ’ phases, while in directionally solidified and equiaxed superalloys it is additionally a MC carbide former. Tantalum is a potential material for high-temperature applications due to its very high melting point (2996 °C), excellent ductility, and unique corrosion properties [30,31]. Consequently, its influence on the microstructure and properties of Inconel 740 for A-USC power plants requires special attention and research. Jablonski [32] indicated that wrought Inconel 740 and its modification Inconel 740H fulfill the quality requirements of A-USC constructions, thus this superalloy in cast form would be very desirable in terms of cost-effectiveness and range of component sizes and geometries. Casting large turbine components with section thickness, even around 100 mm, which weight several tons, requires air casting, as vacuum casting is impractical. When cast in air, aluminum in the nickel alloys oxidizes, leading to aluminum loss from the melt, porosity, and inclusion formation, and so improved air-based casting processes are needed to fulfill the requirements of A-USC power plants components. In this work, two new approaches are presented, namely fabrication of modified alloys based on Inconel 740 in a furnace with argon protection. Chemical composition modifications included increasing Al content and introduction of tantalum. The materials were lost-wax cast with argon shielding to approximate the conditions of the large castings fabrication. The main aim of this work was to investigate the influence of the various Al/Ti ratios and tantalum addition on the primary microstructure, microsegregation of alloying elements, phase transformation temperatures, coefficient of thermal expansion and Vickers hardness.

## 2. Experimental Procedure

### 2.1. Fabrication of Castings

**Figure 1:**
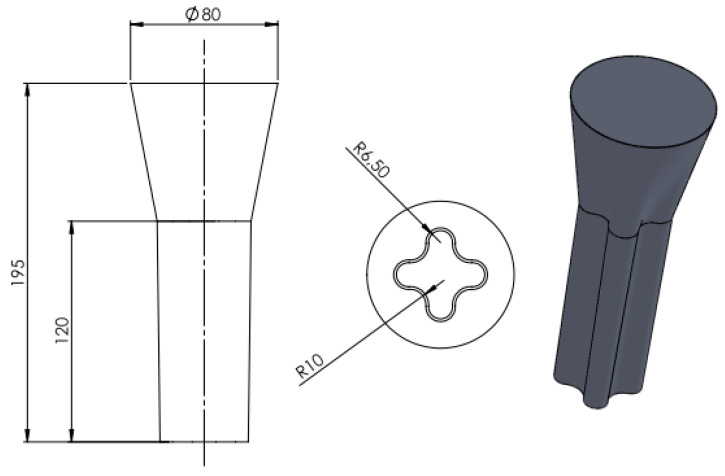
Dimensions of the wax-model.

The casting models and the gating system were made of saturated hydrocarbons (70%—paraffin), fatty acids (20%—stearin) and synthetic waxes (20%—polyethylene wax) mixture. The model mass was prepared in a special tank (equipped with an indirect heating system), where the individual ingredients were melted and mixed in the order: paraffin, stearin, polyethylene wax. Model mass with the required temperature, quality and consistency was obtained by cooling and thorough mixing. Wax models (Figure 1) were obtained by pressing the mass at about 48 °C at 0.5–0.6 MPa in special dies. These models were allowed to cool in the die for about 7 min, and, when solidified, were degreased. Ten molds were fabricated by the “dip and stucco” technique [33] in accordance with data in Table 1. Every layer was formed by dipping the wax pattern into a slurry (binder and filler) and then covered by a coarse dry backup. Preparation of the slurry consisted of mixing in the appropriate proportion of the binder (Ludox PX 30, Sigma-Aldrich, Poznań, Poland) with fine-grained malachite flour. The viscosity of the slurry was tested and used only when its value was appropriate for the requirements of the technological process. After evenly applying a thin coating of the slurry it was covered by a ceramic mixture (43.5 Al_2_O_3_, 53.0 SiO_2_, 1.0 Fe_2_O_3_, 1.6 ThO_2_, 0.1 N_2_O, 0.8 K_2_O; wt%). Stuccoing was carried out by immersing the slurry-coated model in a fluidized layer of sand. For the first and second layers, grain size 0.1–0.3 mm was used, whereas for the rest 0.5–1.0 mm. The established granulation of the quartz sands used was strictly observed. After coating of each layer, the model was air-dried, at 22 °C (±2 °C) and humidity 40–60%, so that the liquid components of the binder (water and ethyl alcohol) evaporated. Drying time for individual layers was between 4 and 12 h, i.e., about 40 h in total. The wax models, covered with 7 layers of the ceramic, were dewaxed in a boilerclave for 20 min. at 140 °C and 4 atm. pressure. Then so-obtained shell molds were burned out at 850 °C for 6 h.

**Figure 2:**
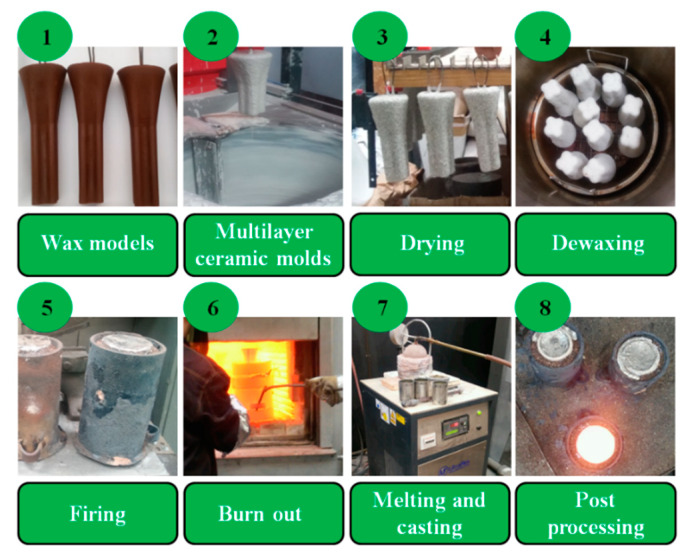
All stages of shell molds and castings preparation.

**Figure 3:**
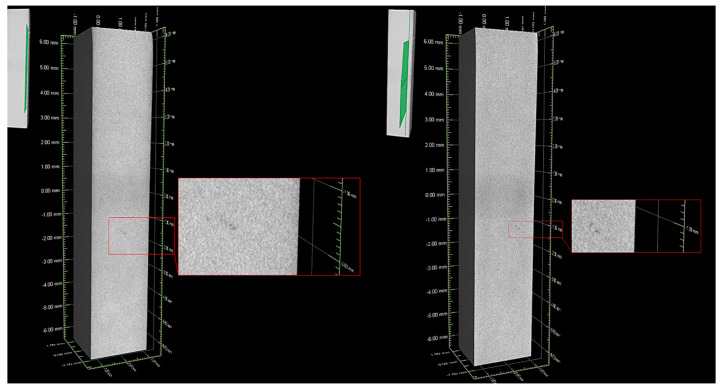
Computed tomography images of the remelted Inconel 740 used for the chemical composition modification.

The 3 kg “master heat” Inconel 740 ingots were melted in argon in a corundum crucible of a UltraMelt S10-SP(S10-SP, UltraFlex Power Technologies, New York, NY, USA) furnace. Melting time was 25 min. and melt-homogenization was 10 min. Double deoxygenating was carried out manually with Ni15Mg compound granules, first after melting the superalloy and secondly, directly before pouring to the preheated mold (1250 °C). For each one 5 g of the compound was used. S-type thermocouples PtRh10-Pt (TER-P-45, ALF-SENSOR, Kraków, Poland) with 0.8 mm wire diameter were used for temperature measurements. After solidification of the molten alloy and ceramic knock-off, the upper part of each casting (75 mm) was cut off and scrapped and the bottom part (120 mm) was used for further investigation. The steps of the casting process are presented graphically in Figure 2. The presence of large porosity within remelted alloy was verified by computed tomography (CT) with GE Phoenix v/tome/x l-450 device (v/tome/x l-450, General Electric, Boston, MA, USA), with acceleration voltage 130 kV, current 55 μA and single voxel size 6.25 μm. The 3D visualization of the casting was reconstructed based on 397 pictures, (Figure 3), with the green surface showing the location of the presented cross-section. Local casting discontinuities were observed, similar in shape to porosities, the size of which did not exceed 30–50 μm. The reconstruction procedure was previously described in detail by Krzak [35].

Remelted Inconel 740 (A0) became the base material for further chemical composition modifications, shown schematically in the Table 2. Aluminum (99.99 purity, rods ϕ6 × 100 mm) and tantalum (99.9 purity, disks with a dimension ϕ10 × 0.2 mm) were added to Inconel 740 in order to obtain nine alloys with three various Al/Ti ratios (0.7, 1.5 and 3.4) and also three tantalum concentrations: 2.0%, 3.0%, and 4.0%. The melting and pouring (1500–1550 °C) processes were carried out similarly to the remelting described above. For each variant, a new crucible was used to prevent undesirable inclusions. To evaluate chemical compositions, three specimens were cut off from the middle part of each casting, ground on sand papers to achieve flat surfaces for the measurement areas, and analyzed using an optical emission spectrometer; the results are presented in Table 3. The concentration relationships of selected alloying elements in the fabricated castings are presented in Table 4.

### 2.2. Preparation of the Samples 

The as-cast specimens (10 × 10 × 10 mm) were cut off from the middle section of each casting and for microscopic examinations were mounted in resin, ground, polished and chemically etched in Aqua Regia (60 mL HCL, 20 mL HNO_3_) for light microscopy (LM) and electrochemically in 10% oxalic acid for scanning electron microscopic (SEM) observations. Using LM images the secondary dendrite arm spacing was measured in 20 various locations. SEM observations were on a Desktop XL instrument with CeB_6_ electron source and the accelerating voltage during SEM-BSE (backscattered electrons) observations and X-ray energy dispersive spectrometry (SEM-EDX) was 20 kV. Segregation coefficient k^i^ = $\frac{C_{D}^{i}}{C_{ID}^{i}}$ (concentration of alloying element “i” in the center of dendrite arm divided by concentration in the interdendritic space determined by SEM-EDX) was calculated, based on the 20 measurements in various locations (each area 10 × 10 μm). The chemical compositions of primary precipitates were also measured by SEM-EDX. Quantitative chemical analysis was carried out using the ZAF correction. In order to calculate the coefficient of thermal expansion (CTE), dilatometric measurements were performed using NETZSCH DIL 402C/4/G device (402C/4/G, NETZSCH, Selb, Germany) on cylindrical samples ϕ6 × 25 mm up to 1200 °C in argon with a heating rate 0.08 °C/s. Differential scanning calorimetry was carried out using a Pegasus DSC 404C/3/G device (404/C/3G, NETZSCH, Selb, Germany) on samples weighting about 40 mg in the range 25–1480 °C with the heating rate 10 °C/min. Hardness testing was performed on the polished samples (at least ten measurements on each sample) using a Zwick/Roell device (Zwick/ZHU187.5, Indentec, West Midlands, UK) with load 10 kgf and 10 s indentation time. 

## 3. Results and Discussion

### 3.1. Characterization of Primary Microstructure by Light Microscopy 

**Figure 4:**
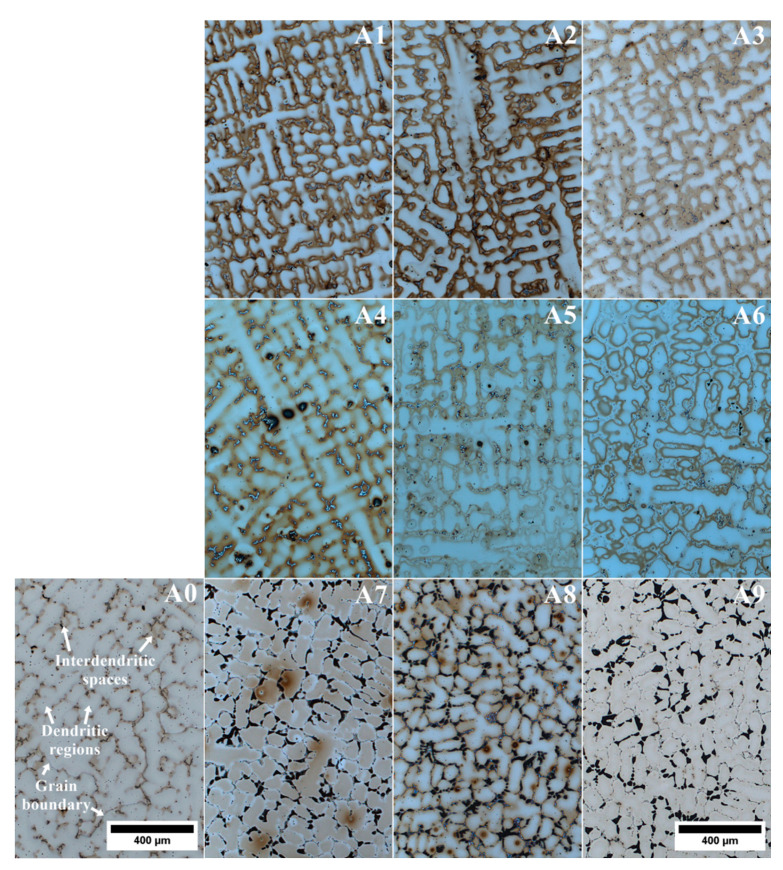
Primary microstructure of remelted Inconel 740 (A0) and modified A1–A9 variants.

**Figure 5:**
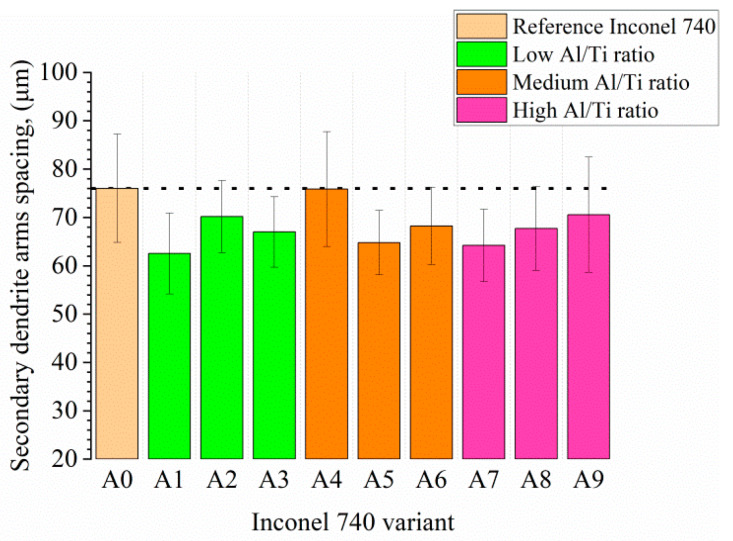
Secondary dendrite arm spacing in fabricated castings.

The microstructure of the remelted Inconel 740 superalloy (A0) and all the variants with aluminium and tantalum (A1–A9) are shown in Figure 4. In all microstructures there were no casting defects, as hot microcracks or misruns. The segregated dendritic microstructure with prime and secondary dendrite arms (SDAS) was visible. The results of SDAS measurements are summarized in Figure 5. The average distance between the secondary dendrite arms in the remelted Inconel 740 was 76 μm, whereas in modified castings it was 63–75 μm. Lack of correlation between SDAS and chemical composition of modified alloys was found. The lowest average value was obtained in A1. In accordance with equation (1) the SDAS (*λ_2_*) is interdependent with the local solidification time (*t_S_*) and rate (*v*), temperature gradient (*G*) and cooling rate [37]. The thermophysical properties of the superalloys and shell molds, the investment casting parameters, and the heat transfer coefficients between the casting and shell mold influence the thermal gradient, the solidification rate, and the cooling rate. Regarding parameters of the casting, it can be the thickness of shell mold, preheating temperature, pouring temperature, and also rate of cooling after solidification. Generally investment casting of Ni-based superalloys is characterized by low cooling rates due to the very low thermal conductivity of shell molds. Matysiak [38] postulated that the cooling rate was around 10–12 °C/min in the temperature range 1263–650 °C, based on investment casting of Inconel 713C. The thermal conductivity of their shell mold (zircon filler, colloidal silica binder, and alumina silicate powders as a back up) was 1.09 W/(mK) at 1200 °C and 0.77 W/(mK) at 600 °C. The pouring was carried out at 1450, 1480, and 1520 °C into preheated (1200 °C) molds. Franke [37] reported considerable differences in the temperature gradient on the surface for thin-walled (width 40 mm, thickness 4 mm) IN738LC superalloy castings. Based on casting’s cross-section, the gradient was 2.75 K/mm on the surface and then reduced with distance from the surface. Ten millimeters from the surface the gradient was 0.25 K/mm, and was constant in the middle part of the casting. Decrease of the gradient from 2.75 to 0.25 K/mm led to an increase of secondary dendrite arm spacing from 30 to 40 μm.

(1) $$\lambda_{2}\sim {\left(t_{s}\right)}^{1/3}\sim {\left(\frac{1}{Gv}\right)}^{1/3}$$

### 3.2. Microsegregation of Alloying Elements Studied by SEM-EDX

**Figure 6:**
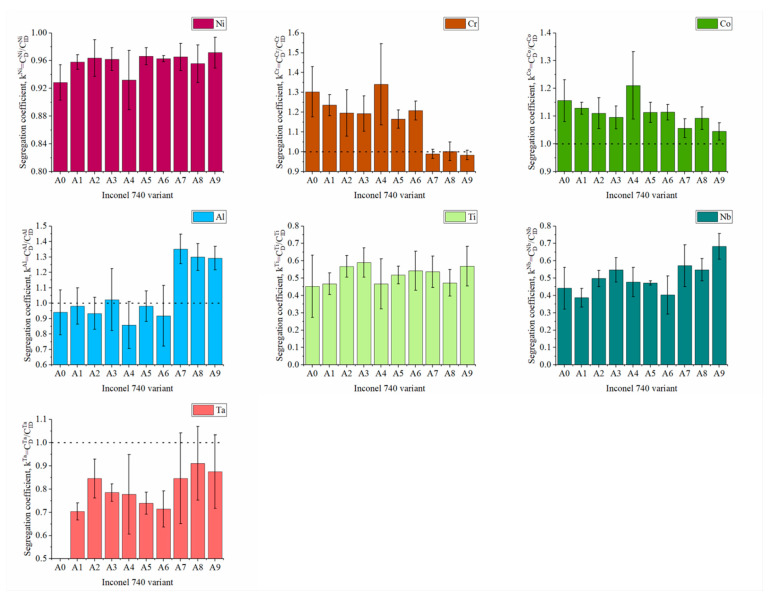
Segregation coefficient k^i^ of alloying elements in the fabricated castings.

Segregation of alloying elements occurring during solidification influenced substantially the large heterogeneity of microstructure. The coefficient k^i^ = $\frac{C_{D}^{i}}{C_{ID}^{i}}$, namely the ratio of the concentration of alloying element in the centerline of dendrite arms to the concentration in the interdendritic spaces, was calculated and is presented in Figure 6, which shows the irregular distribution of elements. Generally, k^i^ is lower than 1 indicating that the element segregated to interdendritic spaces, while when it is greater than 1, the element is more concentrated in the dendritic regions. The most evenly distributed was Ni for which k^Ni^ values were in the range 0.93–0.97. For Cr, k^Cr^ higher than one was obtained in variants A0 to A6, while in A7–A9 the higher Al/Ti ratio did not exceed unity. Cobalt was the only alloying element whose concentration for each superalloy variant was higher in the dendrite arms (k^Co^ < 1). The three highest k_Al_ values were found for superalloys A7–A9 (Al/Ti > 3.0), which were characterized by k^Cr^ < 1. The elements that most strongly segregated to interdendritic spaces were Nb and Ti, for which the coefficients were 0.39 < k^Nb^ < 0.68 and 0.45 < k^Ti^ < 0.59. Similarly to Nb and Ti, Ta concentration was also higher in these spaces and k^Ta^ in each modified variant was below 1.

### 3.3. Characterization of the Primary Microstructure of Castings 

**Figure 7:**
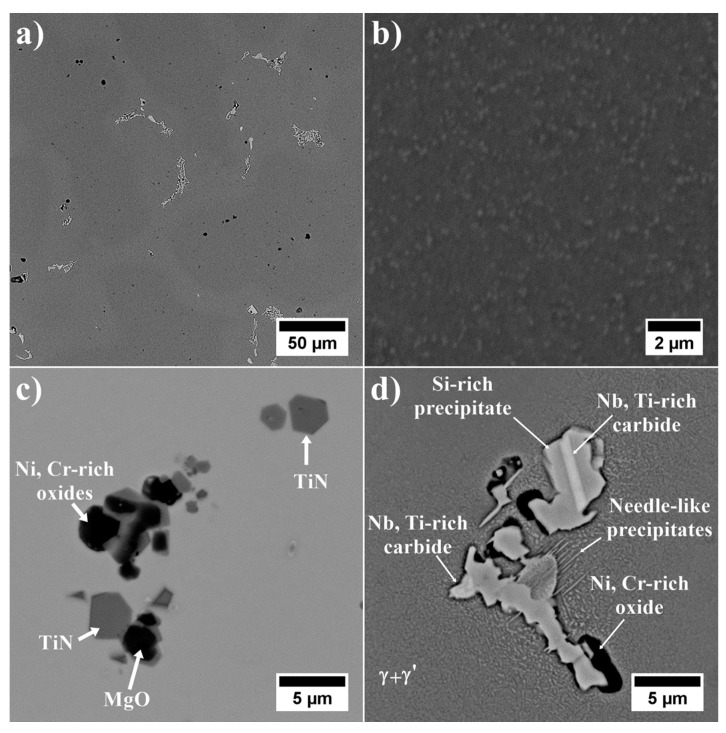
As-cast microstructure of remelted Inconel 740: (**a**) local segregation of microstructural constituents; (**b**) γ’ precipitates in the dendritic region; (**c**) nitrides and oxides in the dendritic region; (**d**) morphology of precipitates in interdendritic spaces.

**Figure 8:**
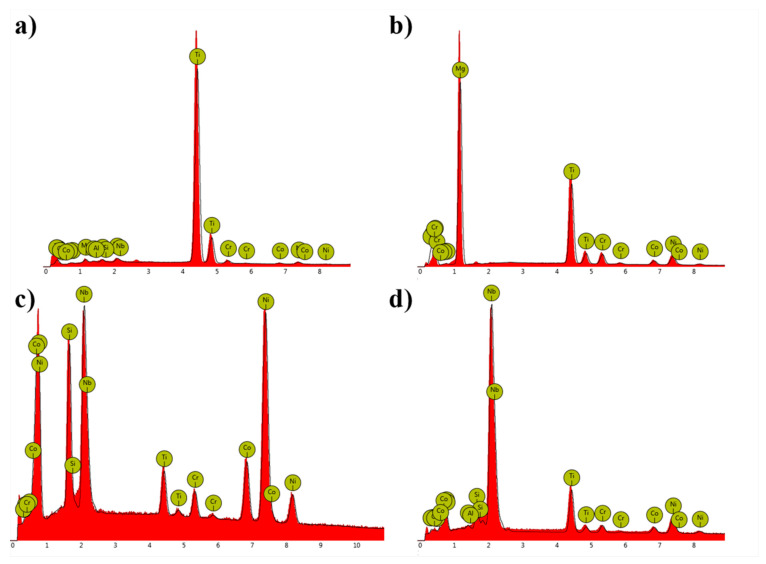
EDS spectra of detected precipitates: (**a**) TiN; (**b**) MgO; (**c**) Si-rich phase; (**d**) (Nb,Ti)C.

SEM microstructures of the remelted Inconel 740 and EDX-spectra of detected precipitates are presented in Figure 7 and Figure 8. The dendritic regions were characterized by a relatively homogeneous microstructure, the γ’ precipitates were surrounded by the matrix. Segregation of the alloying elements during casting solidification led to the formation of many precipitates in the interdendritic spaces. As casting was in argon, locally the Ti-rich nitrides (Figure 8a), MgO (Figure 8b) and also oxides enriched in Ni and Cr were observed. Even in the investment castings produced in vacuum, nitrides and oxides can form [13,38]. Numerous precipitates strongly enriched in Si were also found (Figure 8c). Results obtained by [16,18,39] indicated that Inconel 740 is characterized by the presence of complex silicide, G-phase with face-centered cubic structure (A1, Fm3m) and (M = Nb, Ti)_6_(Ni, Co)_16_Si_7_ formula. It forms on MC carbides or in close vicinity of M_23_C_6_ carbides. G-phase precipitates are undesirable because they lead to a decrease in creep resistance, and therefore their presence in Inconel 740H has been significantly limited. EDX measurements on the precipitates showed the mean chemical composition (at%): Ni-41.9 (±0.5), Si-20.9 (±1.2), Nb-18.4 (±1.7), Co-10.4 (±0.4), Ti-4.1 (±0.6), Cr-4.0 (±2.2). The average relation (M=Nb, Ti)/(Ni, Co)/Si was 7.5/17.4/7. These results were close to the general formula presented above. Evans [39] found a precipitate with a similar composition: Ni-47.3, Si-21.9, Nb-15.3, Co-10.1, Ti-2.8, Cr-2.1, Fe-0.1, Mo-0.2, Al-0.1, which gave a relation (Nb, Ti)/(Ni, Co)/Si proportional to 5.8/18.3/7. Selected area electron diffraction confirmed that it was the G-phase. The same elements can also form a Laves phase, which has the (Ni, Cr, Fe)_2_(Nb, Mo, Ti, Si) formula [40]. According to our EDX results, this relation is 2:1.65, which is mainly affected by the very high Si concentration. Based on EDX measurements of the precipitates in Incoloy 907, it was noticed that the Laves phase does not contain such a high Si content, while, in the study of the Laves phase in Inconel 625, Si was not detected [41,42]. Taking this into account, it can be concluded that Si-rich precipitates detected in the interdendritic spaces probably were complex silicide G-phase.

In accord with Table 3, Inconel 740 contains strong carbide formers. SEM-EDX revealed many precipitates enriched in Nb and Ti (their concentration exceeded 90%), whose Nb/Ti ratio was 3.18 (±0.73) which is typical for the MC-type carbides (Figure 8d). In the close vicinity of the carbides, some needle-like precipitates were observed. Zhao and Evans [16,39] found precipitates with similar morphology in Inconel 740 after long-term annealing, which confirms that it was a DO_24_-ordered hexagonal Ni_3_Ti-η phase. The presence of η phase was also confirmed by Bouse [43] in several as-cast superalloys: Inconel 792+Hf, A286, Inconel 706 and Inconel 901. It indicates that Nb, Ta, Hf and Ti, due to segregation during casting, can to some extent destabilize the desired Ni_3_Al (γ’) phase and promote Ni_3_Ti (η) phase. When the sum of Ti+Nb+Ta exceeds the Al concentration, the formation of η or other platelet phases is favored, either directly from the liquid, or by transformation from the eutectic γ-γ’ islands.

**Figure 9:**
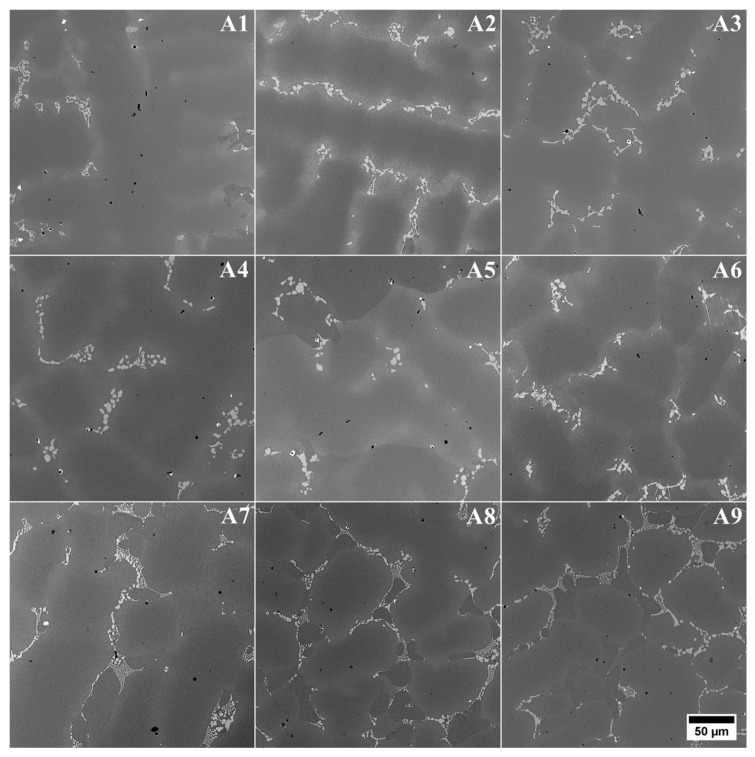
The dendritic structure of the modified alloys.

**Figure 10:**
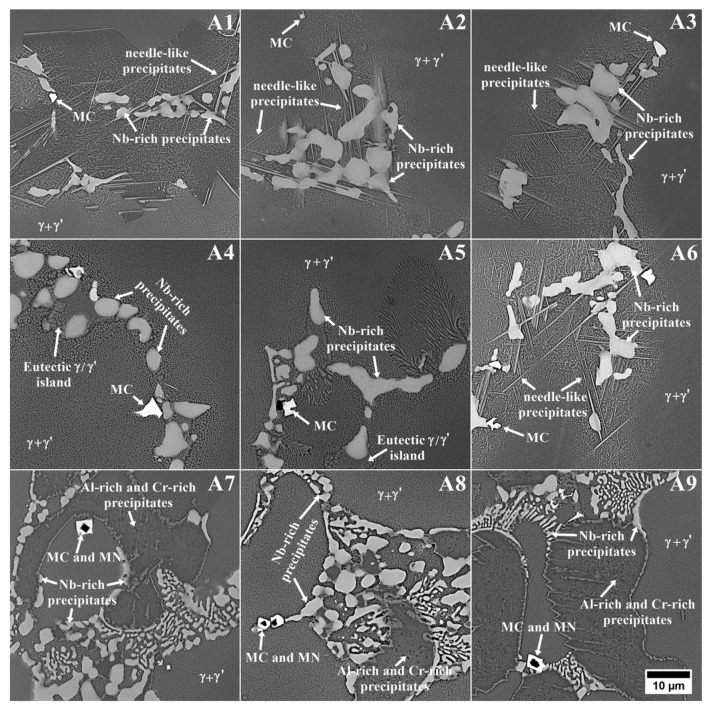
The microstructural constituents in the interdendritic spaces of modified Inconel 740 variants.

**Figure 11:**
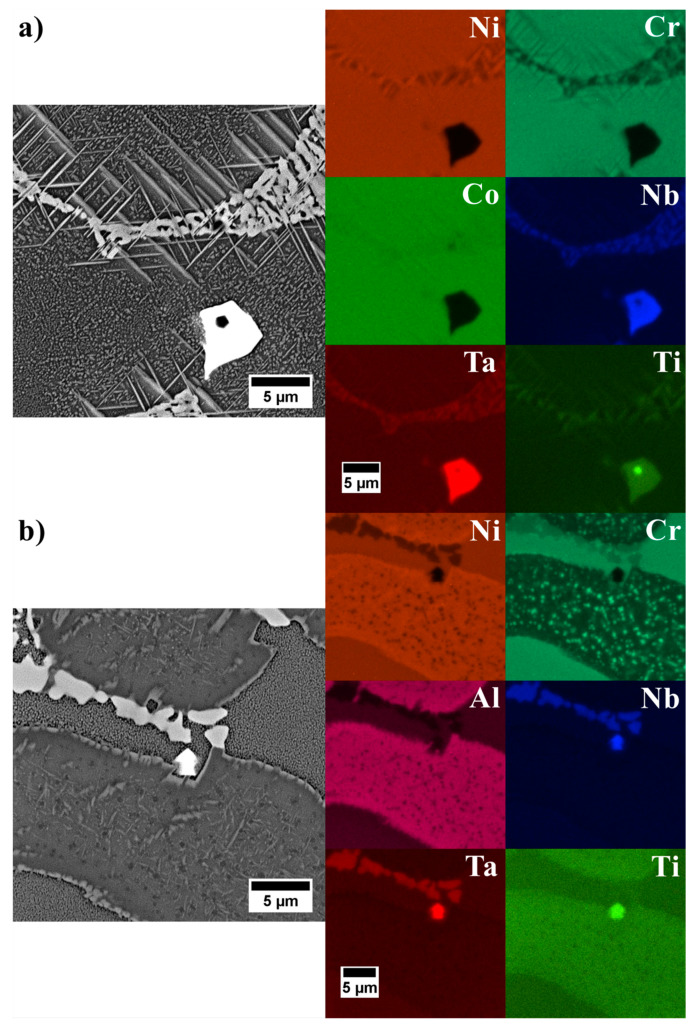
Distribution of selected alloying elements in the interdendritic spaces: (**a**) A1 variant; (**b**) A8 variant.

Modification of Inconel 740 by changing the Al/Ti ratio and introducing tantalum led to major changes in the morphology of primary precipitates (Figure 9). Dendritic regions consisted of fine γ’ precipitates surrounded by the matrix. In the interdendritic regions, the differences were much more complex (Figure 10). Compared to reference A0 alloy (Figure 7a), a significant increase of the needle-like precipitates was observed in alloys A1–A3 and A6. To reveal the differences in the chemical composition of these precipitates with those surrounding, an EDX mapping performed. The A1 variant result (Figure 11a), selected as representative, revealed that needles were enriched in Ti and Ni, which indicated the η phase. The interdendritic region also included Nb-rich precipitates, fine titanium nitrides and, surrounding them, MC carbides with increased Nb, Ti, and Ta concentrations. In A4 and A5 castings, needle-like precipitates were absent, but next to the Nb-rich precipitates, the new components, γ-γ’ eutectic islands were present. Opposite to A4 and A5 alloys with similar Al/Ti ratio, the A6 variant exhibited significant differences in microstructural features. Again the needle-like precipitates appeared, and simultaneously the microstructure was free of γ-γ’ eutectic islands. In the variants A7–A9 with the highest Al/Ti ratios, the changes in microstructure were more pronounced than in A1–A3 and A4–A6 variants in which additional extensive areas with fine precipitates appeared. For more information, EDX analysis was performed in the interdendritic space. Distribution maps from variant 8 with the medium Ta content was chosen as representative. SEM-EDX maps revealed that the areas mentioned earlier were much more enriched in Al than the surrounding dual-phase regions γ matrix + spherical γ’ precipitates (Figure 11b). The presence of such areas with a high concentration of Al results in the segregation coefficient k^Al^ being higher than 1 in variants A7–A9. Aluminum was uniformly distributed in the dendritic region and in the interdendritic spaces strongly Al-rich precipitates were present and the surrounding areas were slightly depleted in Al. In variants A0–A6 such areas were not present and k^Al^ values were around 1, which is usually typical for superalloys. Also, the concentration of Ni was at a higher level there. Numerous fine precipitates within the interdendritic spaces consisted mainly of Cr. Increasing content of strong carbide formers led to formation of carbides in the following order M_7_C_3_→M_23_C_6_→M_6_C→MC [44]. Taking into account the high contents of Nb, Ta and Ti, the probability of numerous Cr-rich carbides is quite low. Based on the tertiary phase diagram Ni-Cr-Al [45], the microstructure and EDX results, it cannot be excluded that such precipitates can be a Cr-rich intermetallic phase surrounded by an intermetallic phase with high Al-content. Presence of numerous Cr-rich precipitates in interdendritic spaces led to the depletion of dendritic regions in Cr, and consequently segregation coefficient k^Cr^ was the lowest in variants A7–A9. Additionally, there were precipitates enriched in Nb and MC carbides consisting mainly of Nb, Ta, and Ti. Titanium nitride has also been detected inside the carbide. 

Chemical composition data of selected precipitates in the interdendritic space were based on EDX measurements at ten different locations in each casting. Table 5 reports the concentration of alloying elements in the Nb-rich precipitates. In variants A1–A3, it was in the range 15.2–17.3%, which gave the ratio Ni/Nb 2.1–2.9. In the group A4–A6, the Nb content increased somewhat to 15.7–17.5% and the Ni content decreased, therefore the Ni/Nb ratio was 1.8–2.3. In the last group A7–A9, the Ni content in precipitates clearly oscillated around 30%, while the Nb concentration slightly increased to 16.7%–19.1%. As a result of these changes, the Ni/Nb ratio was 1.6–2.1. The solidification of Nb-bearing austenitic superalloys like Alloy 718 begins with the formation of Nb-lean austenitic dendrites, while interdendritic eutectic-type solidification constituents involve among others NbC and Nb-rich Laves phase precipitates [46,47,48,49]. In this study, for all modified variants the segregation coefficient of Nb was lower than 1, which confirms that dendritic regions were depleted in this alloying element. Similarities in morphology and composition of the observed precipitates can indicate that they were probably Laves phase. With the increase in the Ta content in superalloys, the Nb/Ta concentration ratio in the precipitates fell. However, this increase was not proportional, which could indicate that Ta was also present in other phases. Baeslack [50] showed that the substitution of Nb by Ta in cast Alloy 718 leads to the formation of TaC and Ta-containing Laves phases. Quantities of Laves phase precipitates in the standard (Ta-free) and Ta-modified superalloys were approximately the same.

**Figure 12:**
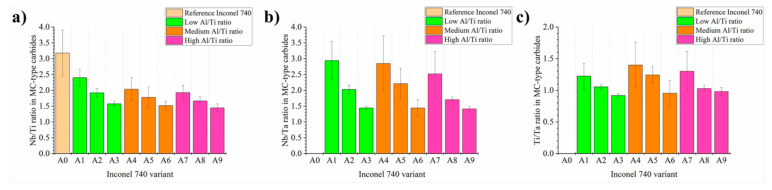
Concentration relationships between selected elements in the MC carbides: (**a**) Nb/Ti; (**b**) Nb/Ta; (**c**) Ti/Ta, based on SEM-EDX results in at%.

EDX maps revealed that generally three elements formed the MC carbides. Their contents are presented as ratios: Nb/Ti, Nb/Ta, and Ti/Ta in Figure 12. Niobium was the main carbide-former both in the reference and the modified alloys. Atom probe tomography conducted by [51] revealed also that Ta can be a γ’-former and, because of its large atomic radius relative to nickel (Ta = 2.00 Å, Ni = 1.49 Å), an effective γ matrix strengthener. Tantalum also, next to niobium and titanium, stabilizes MC-type carbides [44,51,52]. Carbides of alloying elements belonging to the same group show considerable intersolubility, and so the Nb/Ti and Nb/Ta ratios were calculated in order to show the influence of initial superalloy composition on the MC-type carbides composition. The highest Nb/Ti ratio was observed in the reference alloy A0, while in all the modified it was lower, which confirmed the Ta affect on carbides. In each of the groups with low, medium and high Al/Ti ratio, as the initial concentration of Ta increased, the Nb/Ti ratio in carbides fell. Not counting reference A0 superalloy, the highest value of this relation, 2.40, was achieved in variant A1, while the lowest 1.44 in variant A9. Comparison of the Nb/Ta ratios in carbides revealed similarities, as the initial Ta content in the superalloy increased, the value of Nb/Ta ratio decreased. The highest value of 2.94 was achieved in the A1 variant, while the lowest value was 1.41 in A9, although for A3 and A6 the values were quite similar. The last comparison included the change of the Ti/Ta ratio in carbides. Similarly to previous Nb/Ti and Nb/Ta relations, it was observed that in each group (low, medium and high Al/Ti ratio), the increase in the initial Ta content in the superalloy caused a decrease in the Ti/Ta ratio in carbides composition. Changes in these relationships were associated with the partial replacement of niobium (mainly) and Ti by tantalum in the carbides.

### 3.4. Analysis of Coefficient of Thermal Expansion by Dilatometry 

**Figure 13:**
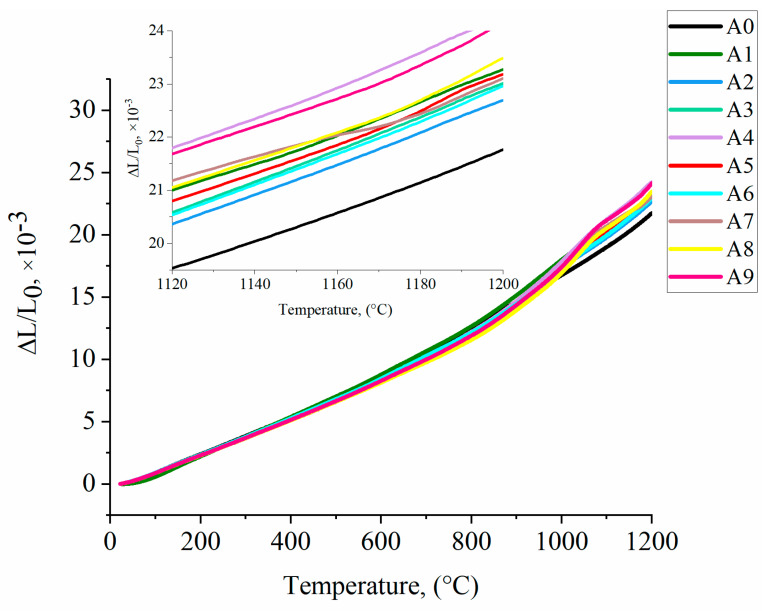
Dilatometric curves for heating at 0.08 °C/s.

Figure 13 shows ∆L/L_0_ as a function of temperature in the range of 25–1200 °C. At 1200 °C, ∆L/L_0_ in each of the modified alloys was clearly higher than in the reference A0, for which it was 21.764 × 10^−3^. The three highest ∆L/L_0_ values, 24.25 × 10^−3^, 24.15 × 10^−3^ and 23.49 × 10^−3^, were observed in variants A4, A9 and A8, respectively. Up to about 800 °C, the ∆L/L_0_ for each of the alloys was increasing linearly, and more rapidly as temperature increased. The gradual slope of A8 and A9 curves around 1000 °C can be related to some secondary reprecipitation from the solid solution. A similar slope was observed in model Ni-Ta-Al-C alloys by Bala [53], who postulated that this dilatometric effect, around 1000 °C, was due to the formation of secondary tantalum carbides. Near this temperature diffusion of tantalum is possible; dissolved primary Ta-rich carbides in the γ matrix being the Ta source. Pyczak [54] reported that the temperature dependence of the average lattice parameters of individual phases γ (a_γ_) and γ’ (a_γ’_) in several superalloys is only similar for lower temperatures. At higher temperatures, a_γ_ starts to increase more rapidly with increasing temperature than a_γ’_. The coefficient of thermal expansion of A0 variant in the range 25–1200 °C was 1.86 × 10^−5^ K^−1^. Among the modified alloys, the highest CTE was 2.07 × 10^−5^ K^−1^(A4), while the lowest was 1.93 × 10^−5^ K^−1^ (A2). All obtained values were shown in Table 6. 

### 3.5. Influence of Chemical Composition on the Solidus and Liquidus Temperatures Studied by DSC 

**Figure 14:**
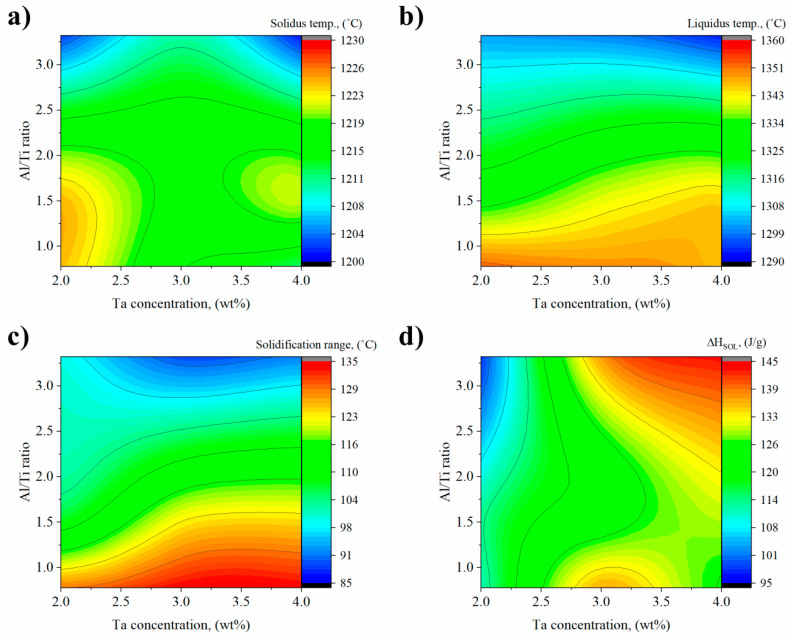
Influence of Al/Ti ratio and Ta concentration in modified alloys on: (**a**) solidus temperature; (**b**) liquidus temperature; (**c**) solidification range; (**d**) enthalpy of solidification.

The solidus (*T_S_*) and liquidus (*T_L_*) temperatures of remelted Inconel 740 estimated by DSC were 1230 °C and 1368 °C, which gave the solidification range 138 °C. The enthalpy of fusion calculated in accordance with

(2) $$\Delta H=\int_{T_{LIQ}}^{T_{SOL}}f\left(T\right)dT$$

was 121.3 J/g. Figure 14 presents the effect of chemical composition modification on the solidus temperature, liquidus temperature, solidification range and enthalpy of melting. Solidus temperature in each of the modified variants was lower than in the reference remelted Inconel 740 (Figure 14a). The highest values among this group (A1–A9) were found in variants A1 and A4, where the beginning of the solid→liquid transformation took place at 1224 °C. It was clearly observed that in A7–A9 alloys with the highest Al/Ti ratios, T_S_ are the lowest. Slight differences in the relationships were observed for the liquidus temperature. Three almost parallel zones related to the Al/Ti ratio are clearly evident on Figure 14b. Similarly to the case of solidus temperatures, the liquidus temperatures were in each modified variant lower than in the reference A0. The highest value 1353 °C was for variant A1, i.e., with the lowest Al/Ti ratio and 2% Ta addition. The increase in the Al/Ti ratio from 0.76 (A1) to 3.35 (A7) at 2% Ta caused the liquidus temperature to decrease by over 50 °C. The difference between the highest (A1) and lowest (A8- only one with T_L_ below 1300 °C) liquidus temperatures was 60 °C. The influence of the chemical composition (Al/Ti ratio and Ta content) on the solidification range shown on Figure 14c also revealed three almost parallel zones similar to liquidus temperatures. The three widest solidification ranges corresponded to the A1–A3 variants, namely with the lowest Al/Ti ratio, 129 °C, 135 °C, and 134 °C, respectively. In alloys with the highest Al/Ti ratio, regardless of the Ta content, the range did not exceed 100 °C. The relationship between chemical composition and resulting enthalpy of solidification revealed that the Al/Ti ratio is not determined, as for *T_S_* and *T_L_*, but the tantalum concentration (Figure 14d). The three lowest ranges were obtained in alloys A1, A4 and A7, i.e., those with a Ta content of 2%, 111.6 J/g, 113.2 J/g and 98.5 J/g, respectively. 

### 3.6. Influence of Chemical Composition Modifications on Hardness 

**Figure 15:**
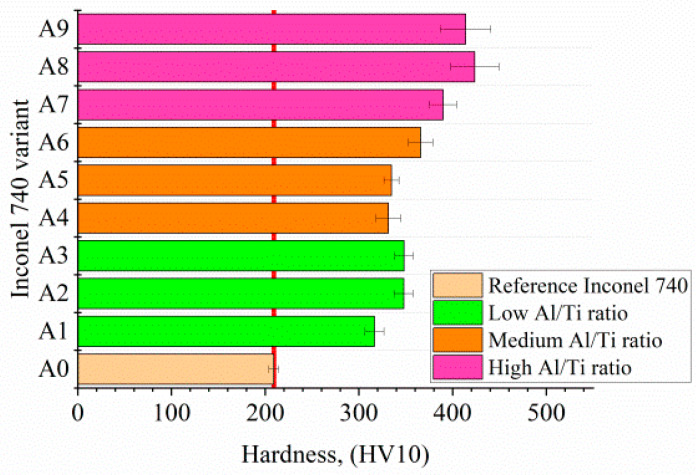
Hardness of the reference Inconel 740 and modified variants.

The average values of Vickers hardness with the standard deviation are shown in Figure 15. The remelted Inconel 740 has hardness 209 HV10, whereas all the modified variants had higher hardness by at least 50%. The hardness of alloys with low Al/Ti ratio was in the range 317–348 HV10. For the alloys with a medium Al/Ti ratio the values were between 331 and 366 HV10, while in those with a high Al/Ti ratio it was 390–423 HV10. 

## 4. Conclusions

Nine newly-developed superalloys based on Inconel 740 were fabricated by investment casting in an induction furnace in argon. The remelted Inconel 740 (A0 variant) was modified by various Al/Ti ratios (0.7, 1.5, 3.4) and Ta concentrations (2.0%, 3.0%, 4.0%). From this work, the following conclusions can be drawn:

- Typical dendritic structures with significant segregation of microstructural constituents to interdendritic spaces were observed in the castings.
- In the remelted Inconel 740, (Nb, Ti)C, TiN, MgO were found. Moreover, phases exhibiting features typical for G-phase and η were detected.
- In all modified castings, the Nb-rich blocky-precipitates, (Nb, Ti, Ta)C carbides, TiN nitrides, oxides, and fine γ’ were observed. In A1–A3 and A6 the Ti-rich needles (probably η phase) were detected, whereas in A4 and A5 variants: eutectic γ-γ’ islands. Variants with the highest Al/Ti ratios had additionally the Al-rich and Cr-rich precipitates.
- With an increase of Ta concentration in each superalloy group (low, medium and high Al/Ti ratio) the Nb/Ti, Nb/Ta and Ti/Ta concentration ratios in carbides decrease gradually.
- In the A0 variant, solidus and liquidus temperatures were higher than in the modified alloys, respectively 1230 °C and 1368 °C. The lowest temperatures observed were in the variants with a high Al/Ti ratio, which also resulted in a narrow solidification ranges, not exceeding 100 °C. With the decrease in Al/Ti, the range of crystallization was wider, even more than 130 °C. These results indicated a much stronger effect of Al than Ta on these phase transformation temperatures.
- All modified variants had higher coefficients of thermal expansion in the temperature range 25–1200 °C. In A0 variant it was 1.86 × 10^−5^ K^−1^, while the highest in A4, 2.07 × 10^−5^ K^−1^.
- Hardness of the reference as-cast Inconel 740 was 209 HV10. By modification of the chemical composition this value increased at least by 50%. The highest hardness value was in A8 variant, 423HV10.

## Figures and Tables

**Table 1:** Procedure of shell mold preparation for the production of modified Inconel 740 superalloys.

Figure 30.	Description						
No of Layer	I	II	III	IV	V	VI	VII
Binder	Ludox PX 30 (synthetic amorphous silica)						
Filler	Molochite flour (Al_2_O_3,_ SiO_2_)						
Ford viscosity (flow cup 6 mm) of slurry, (seconds) [34]	32–35	20–22	22–24			24–26	
Sand grid, (mm)	0.1–0.3		0.5–1				-
Drying time, (hours)	6	4				8–12	

**Table 2:** The modification of Inconel 740 (wt%).

Al/Ti Ratio	Ta Concentration		
	2.0 (±0.10)	3.0 (±0.15)	4.0 (±0.20)
0.7 (±0.10)	A1	A2	A3
1.5 (±0.15)	A4	A5	A6
3.4 (±0.20)	A7	A8	A9

**Table 3:** Nominal composition of Inconel 740, Inconel 740H and limits, according to ASTM requirements, with the nominal values in our modified variants [36].

	Alloy	Cr	Co	Al	Ti	Nb	Fe	Mo	Mn	Si	C	Ta	Ni
Standard	Min.	23.50	15.00	0.20	0.50	0.50	-	-	-	-	0.005	0.00	Balance
	740	24.50	20.00	0.90	1.80	2.00	0.70	0.50	0.30	0.50	0.03	0.00	
	740H	24.50	20.00	1.35	1.35	1.50	0.70	0.50	0.30	0.15	0.03	0.00	
	Max.	25.50	22.00	2.00	2.50	2.50	3.00	1.00	1.00	1.00	0.08	0.00	
Experiment	A0	23.62	20.20	0.90	1.80	1.94	0.63	0.46	0.31	0.62	0.02	0.00	
	A1	22.34	19.96	1.30	1.72	1.80	0.62	0.43	0.30	0.59	0.03	2.00	
	A2	22.46	19.91	1.28	1.78	1.92	0.61	0.43	0.30	0.60	0.02	3.00	
	A3	22.41	19.91	1.31	1.68	1.88	0.62	0.44	0.30	0.61	0.02	4.00	
	A4	22.84	19.71	2.80	1.82	1.92	0.63	0.42	0.29	0.59	0.03	2.00	
	A5	22.74	19.69	2.82	1.76	1.80	0.64	0.43	0.30	0.60	0.03	3.00	
	A6	22.28	19.85	2.75	1.83	1.94	0.56	0.43	0.22	0.56	0.02	4.00	
	A7	21.76	18.85	6.06	1.81	1.88	0.63	0.42	0.29	0.60	0.03	2.00	
	A8	21.20	19.05	6.34	1.76	1.82	0.61	0.40	0.28	0.56	0.03	3.00	
	A9	21.43	18.65	6.12	1.84	1.86	0.62	0.42	0.29	0.60	0.03	4.00	

**Table 4:** Ratios of alloying elements in Inconel 740, Inconel 740H and modified variants.

	Alloy/Ratio	Al/Ti	Al+Ti	Ni/(Al+Ti)	Ta/Al	Ta/Ti
Standard	Min.	0.40	0.70	~86.00	0.00	0.00
	740	0.50	2.70	~18.00	0.00	0.00
	740H	1.00	2.70	~18.00	0.00	0.00
	Max.	0.80	4.50	~8.00	0.00	0.00
Experiment	A0	0.50	2.70	18.33	0.00	0.00
	A1	0.76	3.02	16.20	1.57	1.19
	A2	0.72	3.06	15.60	2.30	1.66
	A3	0.78	2.99	15.70	2.98	2.32
	A4	1.54	4.62	10.16	0.72	1.11
	A5	1.60	4.58	10.07	1.08	1.73
	A6	1.50	4.58	9.94	1.46	2.20
	A7	3.35	7.87	5.80	0.33	1.10
	A8	3.60	8.10	5.56	0.46	1.65
	A9	3.32	7.96	5.55	0.64	2.13

**Table 5:** Chemical composition of Nb-rich precipitates in castings, at%.

Element		Ni	Nb	Cr	Co	Ta	Al	Ti	Ni/Nb	Nb/Ta
Inconel 740 variant	A1	39.3 (±3.0)	15.8 (±2.1)	15.4 (±1.9)	17.2 (±1.1)	5.1 (±2.0)	2.0 (±0.5)	5.1 (±0.9)	2.9 (±0.6)	3.3 (±0.8)
	A2	37.2 (±0.5)	17.3 (±0.6)	15.1 (±0.1)	17.4 (±0.2)	5.7 (±0.3)	2.4 (±0.3)	4.9 (±0.2)	2.1 (±0.1)	3.3 (±0.3)
	A3	37.0 (±1.5)	15.2 (±1.6)	16.4 (±1.2)	17.5 (±0.4)	6.3 (±0.5)	2.7 (±0.8)	5.0 (±0.5)	2.5 (±0.3)	2.4 (±0.2)
	A4	35.1 (±1.4)	17.2 (±1.2)	17.1 (±0.6)	17.5 (±0.4)	3.7 (±1.7)	3.3 (±0.2)	6.1 (±0.9)	1.8 (±0.2)	3.4 (±1.0)
	A5	34.6 (±1.0)	17.5 (±0.4)	16.4 (±0.3)	17.3 (±0.4)	5.2 (±0.3)	4.0 (±0.9)	5.1 (±0.3)	2.0 (±0.1)	3.3 (±0.2)
	A6	36.5 (±1.1)	15.7 (±0.9)	15.9 (±0.4)	17.9 (±0.4)	6.9 (±0.6)	2.5 (±0.7)	4.7 (±0.3)	2.3 (±0.2)	2.3 (±0.4)
	A7	30.2 (±0.9)	19.1 (±0.4)	19.5 (±0.3)	18.2 (±0.5)	4.1 (±0.3)	4.2 (±0.2)	4.7 (±0.5)	1.6 (±0.1)	4.7 (±0.3)
	A8	29.5 (±0.7)	17.2 (±0.3)	19.7 (±0.6)	18.3 (±0.5)	7.5 (±2.2)	3.8 (±0.7)	3.8 (±0.3)	2.1 (±0.1)	2.4 (±0.6)
	A9	29.1 (±0.8)	16.7 (±0.7)	19.6 (±1.1)	18.0 (±0.9)	8.7 (±2.5)	4.3 (±0.6)	3.5 (±0.3)	1.7 (±0.1)	2.0 (±0.5)

**Table 6:** Thermal expansion coefficients of the modified Inconel 740 superalloys.

Variant	A0	A1	A2	A3	A4	A5	A6	A7	A8	A9
Value × 10^−5^, (K^−1^)	1.86	1.98	1.93	1.96	2.07	1.97	1.96	1.97	2.00	2.06

## References

[1] Xie X., Wu Y., Chi C., Zhang M., Aliofkhazraei M. (2015). Superalloys for Advanced Ultra-Super-Critical Fossil Power Plant Application. Superalloys.

[2] Detrois M., Rozman K.A., Jablonski P.D., Hawk J.A. Thermal processing design of cast Inconel Alloy 740H for improved mechanical performance. Proceedings of the 9th International Symposium on Superalloy 718 & Derivatives: Energy, Aerospace, and Industrial Applications.

[3] Ennis P. (2014). Nickel-Base Alloys for Advanced Power Plant Components. Coal Power Plant Materials and Life Assessment.

[4] Rakoczy Ł., Grudzień M., Tuz L., Pańcikiewicz K., Zielińska-Lipiec A. (2017). Microstructure and properties of a repair weld in a nickel based superalloy gas turbine component. Adv. Mat. Sci..

[5] Abbasi M., Kim D., Shim J., Jung W. (2016). Effect of alloyed aluminum and titanium on the oxidation behavior of Inconel 740 superalloy. J. Alloys Compd..

[6] Pirowski Z. (2013). Nickel Alloys as a Modern Casting Material for Operation in Extreme Operating Conditions.

[7] Abstoss K.G., Schmigalla S., Schultze S., Mayr P. (2019). Microstructural changes during creep and aging of a heat resistant MARBN steel and their effect on the electrochemical behaviour. Mat. Sci. Eng. A.

[8] Rakoczy Ł., Grudzień M., Cygan R., Zielińska-Lipiec A. (2019). Effect of cobalt aluminate content and pouring temperature on macrostructure, tensile strength and creep rupture of Inconel 713 castings. Arch. Metall. Mater..

[9] Rakoczy Ł., Grudzień M., Cygan R. (2019). Influence of melt-pouring temperature and composition of primary coating of shell mold on tensile strength and creep resistance of Ni-based superalloy. J. Mat. Eng. Perform..

[10] Dudziak T., Deodeshmukh V., Backert L., Sobczak N., Witkowska M., Ratuszek W., Chruściel K., Zieliński A., Sobczak J., Bruzda G. (2017). Phase investigation under steam oxidation process at 800 °C for 1000 h of advanced steels and Ni-based alloys. Oxid. Met..

[11] Rakoczy Ł., Grudzień-Rakoczy M., Cygan R. (2019). The influence of shell mold composition on the as-cast macro- and micro-structure of thin-walled IN713C superalloy casting. J. Mat. Eng. Perform..

[12] Hanning F., Khan A.K., Steffenburg-Nordenström J., Ojo O., Andersson J. (2019). Investigation of the effect of short exposure in the temperature range of 750–950 °C on the ductility of Haynes^®^ 282^®^ by advanced microstructural characterization. Metals.

[13] Matysiak H., Zagorska M., Andersson J., Balkowiec A., Cygan R., Rasinski M., Pisarek M., Andrzejczuk M., Kubiak K., Kurzydlowski K.J. (2013). Microstructure of Haynes^®^ 282^®^ superalloy after vacuum induction melting and investment casting of thin-walled components. Materials.

[14] Wilk-Kołodziejczyk D., Regulski K., Giętka T., Gumienny G., Jaśkowiec K., Kluska-Nawarecka S. (2018). The selection of heat treatment parameters to obtain austempered ductile iron with the required impact strength. J. Mat. Eng. Perform..

[15] Di Gianfrancesco A. (2017). Materials for Ultra-Supercritical and Advanced Ultra-Supercritical Power Plants.

[16] Zhao S.Q., Xie X.S., Smith G.D., Patel S.J. (2003). Microstructural stability and mechanical properties of a new nickel-based superalloy. Mater. Sci. Eng. A.

[17] Baker B.A. A new alloy designed for superaheater tubing in coal-fired ultra supercritical boilers. Proceedings of the International Symposium on Superalloys 718, 625, 706 and Derivatives, TMS (The Minerals, Metals & Materials Society).

[18] Unocic K.A., Shingledercker J.P., Tortorelli P.F. (2014). Microstructural changes in Inconel 740after long-term aging in the presence and absence of stress. JOM.

[19] Rai A.K., Tripathy H.P., Hajra R.N., Raju S., Saibaba S., Jayakumar T. (2016). Measurement of high temperature phase stability and thermophysical properties of alloy 740. Mat. Sci. Technol..

[20] Zhao S.G. (2006). Experimental investigation and thermodynamic calculation on phase precipitates of Inconel 740. Acta Metall. Sin..

[21] Grudzień M., Cygan R., Pirowski Z., Rakoczy Ł. (2018). Microstructural characterization of Inconel 713C superalloy after creep testing. Trans. Found. Res. Inst..

[22] Rozman K.A., Detrois M., Jablonski P.D., Hawk J.A. Mechanical performance of various Inconel^®^740/740H alloy compositions for use in A-USC castings. Proceedings of the 9th International Symposium on Superalloy 718 & Derivatives: Energy, Aerospace, and Industrial Applications.

[23] Chong Y., Liu Z., Godfrey A., Wang L., Liu W., Weng Y. (2015). Heat treatment of a candidate material for 700 °C A-USC power plants. J. Iron Steel. Res. Inter..

[24] Sroka M., Zieliński A., Hernas A., Kania Z., Tański T., Śliwa A. (2017). The effect of long-term impact of elevated temperature on changes in the microstructure of Inconel 740H alloy. Metalurgija.

[25] Zieliński A., Sroka M., Dudziak T. (2018). Microstructure and mechanical properties of Inconel 740H after long-term service. Materials.

[26] Shingledecker J.P., Evans N.D., Pharr G.M. (2013). Influences of composition and grain size on creep-rupture behavior of Inconel^®^alloy 740. Mat. Sci. Eng. A.

[27] Zhao S., Xie X., Smith G.D., Patel S.J. (2006). Research and Improvement on structure stability and corrosion resistance of nickel-base superalloy Inconel alloy 740. Mat. Design..

[28] Shin G.S., Yun J.Y., Park M.C., Kim S.J. (2014). Effect of Mo on the thermal stability of γ’ precipitate in Inconel 740 alloy. Mat. Char..

[29] Xie X., Zhao S., Dong J., Smith G.D., Baker B.A., Patel S.J. (2007). Modification of Ni-Cr-Co-Mo-Nb-Ti-Al superalloy for USC power plant application at temperature above 750°C. Mat. Sci. Forum..

[30] Buckman R.W. (2000). New applications for tantalum and tantalum alloys. JOM.

[31] Janowski G.M., Heckel R.W., Pletka B.J. (1986). The effects of tantalum on the microstructure of two polycrystalline nickel-base superalloys: B-1900 + Hf and MAR-M247. Metall. Trans. A.

[32] Jablonski P.D., Hawk J.A. Inconel^®^ Alloy 740: Potential for use in A-USC castings. Proceedings of the 8th International Symposium on Superalloy 718 & Derivatives: Energy, Aerospace, and Industrial Applications.

[33] Angrecki M., Kamińska J., Jakubski J., Wieliczko P. (2019). Strength properties of ceramic moulds containing spent moulding sand after initial reclamation. Arch. Found. Eng..

[34] ISO (2019). 2431:2019 Paints and Varnishes—Determination of Flow Time by Use of Flow Cups.

[35] Krzak I., Tchórz A., Pirowski Z., Jaśkowiec K., Grudzień M., Purgert R. (2018). Quality check of H282 castings by Computed Tomography (CT) technique. Trans. Found. Research Inst..

[36] ASTM (2017). B983—16E1 Standard Specification for Precipitation Hardened or Cold Worked, Seamless Nickel Alloy Pipe and Tube.

[37] Franke M.M., Hilbinger R.M., Konrad C.H., Glatzel U., Singer R.F. (2011). Numerical determination of secondary dendrite arm spacing for IN738LC investment casting. Metall. Trans. A.

[38] Matysiak H., Zagorska M., Balkowiec A., Adamczyk-Cieslak B., Dobkowski K., Koralnik M., Cygan R., Nawrocki J., Cwajna J., Kurzydłowski K. (2016). The influence of the melt-pouring temperature and inoculant content on the macro and microstructure of the IN713C Ni-based superalloy. JOM.

[39] Evans N.D., Maziasz P.J., Swindeman R.W., Smith G.D. (2004). Microstructure and phase stability in Inconel alloy 740 during creep. Scr. Mat..

[40] Rozmus-Górnikowska M., Blicharski M. (2015). Microsegregation and precipitates in Inconel 625 arc weld overlay coatings on boiler pipes. Arch. Metall. Mat..

[41] Petrzak P., Kowalski K., Blicharski M. (2016). Analysis of phase transformations in Inconel 625 alloy during annealing. Acta Phys..

[42] Baeslack W.A., Ernst S.C., Lippold J.C. (1989). Weldability of high-strength, low-expansion superalloys. Weld. J..

[43] Bouse G.K. Eta (η) and Platelet Phases in Investment Cast Superalloys. Proceedings of the 8th International Symposium Superalloys 1996.

[44] Jena A.K., Chaturvedi M.C. (1984). The role of alloying elements in the design of nickel-base superalloys. J. Mat. Sci..

[45] Raghavan V. (2008). Al-Cr-Ni (Aluminum-Chromium-Nickel). J. Phase Equilibria Diffus..

[46] Sohrabi M.J., Mirzadeh H., Rafiei M. (2018). Solidification behavior and Laves phase dissolution during homogenization heat treatment of Inconel 718 superalloy. Vacuum.

[47] Rakoczy Ł., Cygan R. (2018). Analysis of temperature distribution in shell mould during thin-wall superalloy casting and its effect on the resultant microstructure. Arch. Civ. Mech. Eng..

[48] Shi X., Duan S., Yang W., Guo H., Guo J. (2018). Solidification and segregation behaviors of superalloy IN718 at a slow cooling rate. Materials.

[49] Knorovsky G.A., Cieslak M.J., Headley T.J., Romig A.D., Hammetter W.F. (1989). Inconel 718: A solidification diagram. Metall. Trans. A.

[50] Baeslack W.A., West S.L., Kelly T.J. (1988). Weld cracking in Ta-modified cast Inconel 718. Scr. Metall..

[51] Reed R.C., Yeh A.C., Tin S., Babu S.S., Miller M.K. (2004). Identification of the partitioning characteristics of ruthenium in single crystal superalloys using atom probe tomography. Scr. Mat..

[52] Rakoczy Ł., Grudzień M., Zielińska-Lipiec A. (2018). Contribution of microstructural constituents on hot cracking of MAR-M247 nickel based supealloy. Arch. Met. Mat..

[53] Bała P. (2012). Ni-Ta-Al-M Alloys with High Carbon Concentration.

[54] Pyczak F., Devrient B., Mughrabi H. (2004). The effects of different alloying elements on the thermal expansion coefficients, lattice constants and misfit of nickel-based superalloys investigated by X-ray diffraction. Superalloys.

